# A Simple Remedy for Sprains

**Published:** 1892-10

**Authors:** 


### A SIMPLE REMEDY FOR SPRAINS, ETC.

Many people still rely upon wormwood and rum, arnica and other applications in the form of liniments in the treatment of bruises and sprains, and ignore that simple but most efficient remedy of all, hot water. There is no medicine known that approaches it in value in injuries such as these in which the skin is not broken.

If a train of cars is derailed, wreckers are sent out at once from headquarters ; and the greater the number the sooner their work is done. The blood may not improperly be likened to these men, for it is nature's means of repair, and when an injury occurs she immediately diverts to the affected parts a large supply of blood freighted with the needed materials. In some instances, of course, supply is much greater than is actually required, and the work of restoration is in consequence obstructed, but the excess is practically made up of lookers on, or interlopers, as it were, for which nature is not responsible.

The ball field seems to be very favorable for bruises and sprains, and one would naturally suppose professionals, at least, would be well up in the most approved form of treatment. Players, however, are often disabled for weeks by hurts that ought to be repaired in a few days.

If an injury is so located treatment can be conveniently applied, as on the hand, the arm, or leg, at once after receiving it, the affected parts should be plunged into water as hot as can be borne, and kept there for several hours. When the bath must be discontinued the injured parts should have water dressings applied, and these in turn be kept hot until all the tenderness has been drawn out.

Severe bruises, wrenches. and sprains, when treated in this way, are far more quickly cured than by any other known means. And the sufferings of the patients are infinitely less than where liniments, lotions, etc., are used. In such injuries, after the soreness has been relieved. there is generally some swelling or puffiness of the parts to overcome.

